# Sweat analysis with a wearable sensing platform based on laser-induced graphene

**DOI:** 10.1063/5.0093301

**Published:** 2022-09-19

**Authors:** F. Vivaldi, A. Dallinger, N. Poma, A. Bonini, D. Biagini, P. Salvo, F. Borghi, A. Tavanti, F. Greco, F. Di Francesco

**Affiliations:** 1 Department of Chemistry and Industrial Chemistry, University of Pisa, via Giuseppe Moruzzi 13, 56124 Pisa, Italy; 2 Institute of Clinical Physiology, National Research Council, via Giuseppe Moruzzi 1, 56124 Pisa, Italy; 3 Institute of Solid State Physics, NAWI Graz, Graz University of Technology, 8010 Graz, Austria; 4 Department of Biology, University of Pisa, 56127 Pisa, Italy; 5 Interdisciplinary Center for Nanostructured Materials and Interfaces, Department of Physics, University of Milan, Via Celoria 16, Milan 20133, Italy; 6 The Biorobotics Institute, Sant'Anna School of Advanced Studies, Viale R. Piaggio 34, 56025 Pontedera, Italy; 7 Department of Excellence in Robotics and AI, Sant'Anna School of Advanced Studies, P.zza Martiri della Libertà, 56127 Pisa, Italy

## Abstract

The scientific community has shown increasing interest in laser scribing for the direct fabrication of conductive graphene-based tracks on different substrates. This can enable novel routes for the noninvasive analysis of biofluids (such as sweat or other noninvasive matrices), whose results can provide the rapid evaluation of a person's health status. Here, we present a wearable sensing platform based on laser induced graphene (LIG) porous electrodes scribed on a flexible polyimide sheet, which samples sweat through a paper sampler. The device is fully laser manufactured and features a two layer design with LIG-based vertical interconnect accesses. A detailed characterization of the LIG electrodes including pore size, surface groups, surface area in comparison to electroactive surface area, and the reduction behavior of different LIG types was performed. The bare LIG electrodes can detect the electrochemical oxidation of both uric acid and tyrosine. Further modification of the surface of the LIG working electrode with an indoaniline derivative [4-((4-aminophenyl)imino)-2,6-dimethoxycyclohexa-2,5-dien-1-one] enables the voltammetric measurement of pH with an almost ideal sensitivity and without interference from other analytes. Finally, electrochemical impedance spectroscopy was used to measure the concentrations of ions through the analysis of the sweat impedance. The device was successfully tested in a real case scenario, worn on the skin during a sports session. In vitro tests proved the non-cytotoxic effect of the device on the A549 cell line.

## INTRODUCTION

The unobtrusive remote monitoring of health conditions is key in biomedical applications. In fact, aging populations are increasing the pressure on national health care systems, in terms of the demand for innovative services that both improve the quality of life and financial sustainability.^1^ Managing patients at home and reducing the hospital workload is one of the main ways of meeting these goals through new reliable sensing technologies. These sensors can be used on a continuous basis to measure the (bio)chemical parameters of a number of biofluids that can be collected unobtrusively, such as saliva, sweat, and tears. Although blood analyses play a key role in modern medicine, blood collection is limited in terms of invasivity, production of hazardous waste, and the need for trained personnel. Blood collection is, thus, not compatible with portable or wearable sensing devices managed by nonprofessional users. However, blood concentrations of some drugs or biomarkers are correlated with concentrations in biological fluids such as saliva, tears, and sweat that can be collected unobtrusively by simple systems.^2–6^ Sweat is a model fluid for testing new sensing technologies as it is produced during physical activity over a large surface area and it contains physiologically significant chemicals such as glucose, lactate, urea, uric acid,^7^ amino acids (e.g., tyrosine), and electrolytes (e.g., sodium, potassium, and chloride).^8–10^ Uric acid, for example, can provide insight concerning the oxidative stress^11^ generated in an athlete during physical activity or disorders such as chronic renal disease.^12,13^ Tyrosine supplements seem to improve the exercise capacity in heat,^14^ but abnormal concentrations have been related to metabolic disorders^15,16^ and liver disease.^17^ Sweat conductivity results from the overall ionic concentration and mirrors the hydration status of the subject and the loss of the main electrolytes during exercise.^18,19^ Since sweat is a buffered solution, variation in its pH may be directly associated with major diseases.^20,21^

This interest in sweat analysis is confirmed by recent literature. Wei *et al.*^22^ developed a carbon-based electrode made up of electrospun carbon fibers in order to measure uric acid in sweat. The large surface area of fibers granted by many active sites foster the transfer of electrons produced in the oxidation of uric acid. Yang *et al.*^23^ proposed a device for the determination of tyrosine and uric acid in sweat by conductive tracks engraved on a polyimide (PI) sheet. A wireless wearable system measuring sweat pH was proposed by Mazzaracchio *et al.*;^24^ however, a multiparametric analysis is still lacking due to the complexity of multiple transduction and possible cross-interference effects.

This work describes the fabrication, calibration, and testing of a device combining an unobtrusive paper sweat sampler and laser induced graphene (LIG) electrodes engraved on a thin, flexible polyimide sheet. Laser-induced pyrolysis allows the direct fabrication of conductive porous carbon tracks on polymeric substrates.^25^ The high surface area and conductivity (∼25 S/cm^26^) make LIG a promising material for the fabrication of tracks and electrodes in the field of printed/flexible electronics and wearable electrochemical sensors.^27^ The LIG morphology, specific surface area, wettability, conductivity, and chemical composition can be tuned by either changing the lasering parameters,^26,28–30^ the environment,^31^ or the polymer precursor.^32^ Compared to traditional techniques (e.g., screen and inkjet printing), laser scribing does not need additional chemicals to tune the surface properties, and the transition to green manufacturing is possible through the selection of suitable bio-based precursor materials.^33–37^

In this work, the LIG working electrode was functionalized with an indoaniline derivative 4-((4-aminophenyl)imino)-2,6-dimethoxycyclohexa-2,5-dien-1-one (IAd), undergoing a reversible electrochemical reaction characterized by a negative pH-dependent reduction potential. Since the redox potentials of uric acid (UA) and tyrosine are external to the IAd potential window, the same electrode could be used for an almost simultaneous analysis of the three analytes. Square wave voltammetry (SWV) was used as the electrochemical transduction technique for the rapid detection of the peak position of the IAd and to measure the current at the oxidation peaks of UA and tyrosine. The same electrochemical setup was used to monitor sweat resistance through electrochemical impedance spectroscopy (EIS) and was related to the concentration of the electrolytes.

## RESULTS AND DISCUSSION

### Characterization of LIG

The basis for the electrochemical platform for noninvasive biofluid analysis consisted of a polyimide (PI) sheet with laser scribed LIG electrodes and tracks.

Preliminary investigations concerned the performance of two LIG species with different morphologies, a porous (LIG-P) type and a fibrous (LIG-F) type, created by setting different laser scribing parameters.^29^ Figure S1 shows SEM images of the morphology and cyclic voltammograms in the presence of 5 mM K_4_Fe(CN)_6_ for both bare/reduced LIG-P and LIG-F. Despite the higher current densities, the larger variation between the bare and reduced LIG-F and the reduction peak at −1.2 V (Fig. S2) suggest a higher concentration of surface impurities, which are likely a consequence of the higher laser fluence used to scribe this variant.^26^ These preliminary results and the fragility of the fibers pointed to LIG-P being the best species for sensor fabrication, as also concluded by Behrent *et al.*^38^

**FIG. 1.**
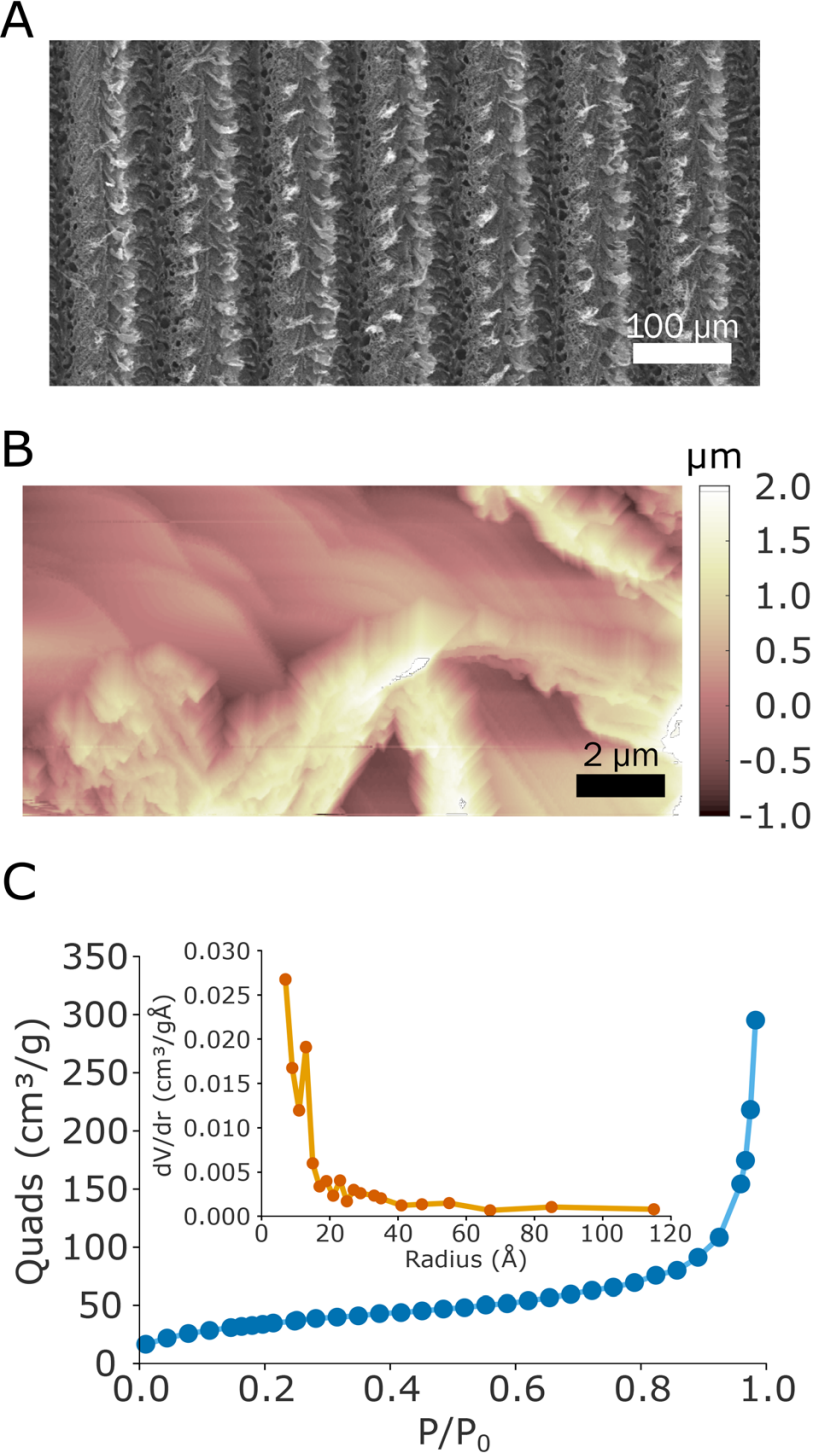
(a) SEM image of the scribed LIG electrodes. (b) AFM topographic image of the LIG inside a laser scribed trench. (c) Nitrogen adsorption isotherm of LIG. The inset shows the corresponding pore size distribution calculated by the BJH method.

An extensive investigation of the LIG-P morphology (hereinafter LIG) was carried out. The SEM image [Fig. 1(a)] shows the corrugated structure of LIG and the trenches created by the laser scribing. The AFM image [Fig. 1(b)] acquired in the middle of a trench also shows a very rough and disordered LIG surface with micrometer high peaks and valleys. This corrugated morphology increases the surface area at the interface. The specific surface area, calculated by the curve describing the quantity of nitrogen adsorbed by increasing its pressure [adsorption isotherm in Fig. 1(c)], is A_LIG_ = (126 ± 11) m^2^/g. The inset in Fig. 1(c) shows the pore size distribution (calculated as in the Methods section) with little or no signs of mesopores but the presence of micropores in the LIG structure.

**FIG. 2.**
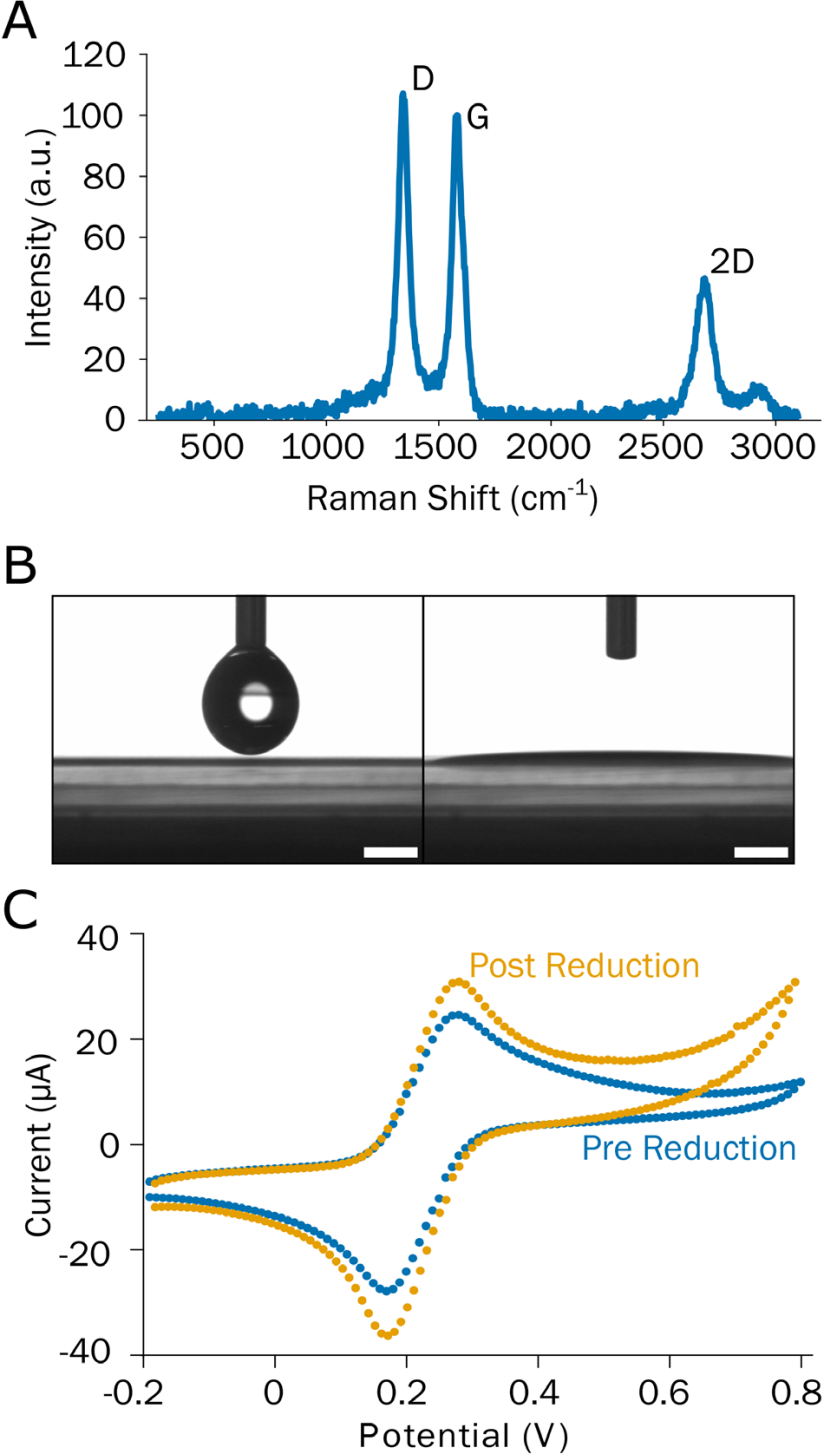
(a) Raman spectrum showing the typical bands of LIG. (b) Contact angle measurement of LIG showing a superhydrophilic behavior (scale bar = 1 mm). (c) Cyclic voltammetry performed on LIG in the presence of ferrocyanide 5 mM, before and after electrochemical reduction in 0.1 M KCl.

In order to characterize the composition and structural properties of LIG, Raman spectroscopy was performed. The Raman spectrum [Fig. 2(a)] shows the typical bands (D, G, and 2D) found in LIG. The D-band (v_d_ ∼1350 cm^−1^) is associated with the disorder of the graphene lattice and shows that LIG has many defects (five- and seven-carbon atom rings). The ratio of the D- and G-band, I(D)/I(G) = 1.06, also indicates a large number of defects. The 2D-band (v_2D_ ∼ 2700 cm^−1^) clearly indicates the presence of a crystalline (graphitic) structure.

The study of the wettability of LIG, which shows a contact angle of φ = 0° [Fig. 2(b)], highlights its superhydrophilicity, which may arise from the oxygen atoms on the surface, as confirmed by XPS data (Fig. S3 in the supplementary material), and also from the high corrugation/porosity of the LIG structure. An atomic oxygen content of 5% was calculated for the LIG from XPS measurements, which agrees with the findings of Li *et al.*^31^ for hydrophilic LIG.

The sheet resistance of LIG was measured as R_◻_ = 43 ± 1 Ω/◻, with a thickness of d_LIG_ = 10 ± 2 *μ*m measured by the profilometer. Assuming a homogeneous filling (which is unlikely), this would result in a conductivity of σ_LIG_ = 23 ± 5 S/cm. Note that this represents a largely underestimated value, because of the very low density and highly porous structure.

Figure 2(c) shows the performance of freshly prepared and reduced LIG electrodes with an electroactive probe (ferrocyanide K_4_Fe(CN)_6_ 5 mM) and highlights how the reduction treatment increases the peak current. In a reversible electrochemical reversible reaction, this parameter can be described by the Randles–Sevcik equation^39^ as follows:

$$i_{p}=0.4463\;\mathrm{nFAC}{\left(\frac{\mathrm{nFvD}}{RT}\right)}^{1/2},$$ (1)

where n is the number of electrons, F is the Faraday constant, A is the electroactive area, C is the concentration of the electroactive probe, v is the scan rate, D is the diffusion coefficient, R is the ideal gas constant, and T is the temperature. Consequently, the higher current can be explained as an increase in the electroactive area of the LIG surface. The scan rate analysis performed in a 0.1 M KCl solution with 5 mM K_4_Fe(CN)_6_ enabled us to calculate electroactive areas of 0.050 ± 0.003 and 0.02 ± 0.01 cm^2^ (n = 5) for reduced and bare LIG, respectively. For this electrolyte/electroactive probe combination, a value of 0.65 × 10^−5^ cm^2^/s was used for the diffusion coefficient of Fe(CN)_6_^4−^ as reported in Ref. 40.

The reduction removes the oxygenated groups from the LIG surface, as shown from the cyclic voltammogram of LIG electrodes in 0.1 M KCl (Fig. S4), where a reduction peak with decreasing intensity among different cycles (irreversible process) is visible at −1.2 V vs AgCl as for graphene oxide.^41^ The need for a reduction treatment with many types of LIG electrodes^42^ is related to the impurities of LIG derived from the decomposition process and ambient atmosphere. Despite the increase in the electroactive area after the reduction process, this average value was barely higher than the geometric area (A_electroactive_/A_geom_ = 1.61), which is in contrast with the surface area calculated by the nitrogen adsorption analysis (A_N2_/A_geom_ ≈ 250). In line with Behrent *et al.*,^38^ such results can be justified by assuming that the electroactive probe may not be able to penetrate the pores, which consequently reduces the overall available surface for the electrochemical reaction. This hypothesis is in agreement with the absence in LIG of meso and macro-pores (smaller than a few hundreds of nanometers) resulting from nitrogen adsorption measurements [Fig. 1(c)] and the presence of just a micropore structure. This is a common phenomenon observed with LIG electrodes, although examples of LIG with higher surface ratios (A_electroactive_/A_geom_ = 18.5) have been reported.^43^ However, there is no reliable explanation for the different surface values estimated from the electroactive area and nitrogen adsorption.^44^

Wearing the device on skin induces a mechanical stress on the sensor that was simulated by 100 bending/relaxation cycles (bending radius = 7 mm) in electromechanical tensile/compression testing equipment. In the case of LIG, track resistance only increased by ∼2% [Fig. S5(a)]; thus, this material showed a good stability against wearing stress, whereas the resistance of the LIG/Ag tracks increased by ∼10% [Fig. S5(b)], likely due to fractures in the LIG/Ag composite caused by the mechanical stress. However, the resistance of pristine LIG/Ag tracks (R_0_ = 50 Ω) was over 30 times smaller than the resistance of LIG tracks, which is a major improvement.

### Characterization of the sensor platform

The two layer sensor design with VIAs (see details in Methods section) was adopted to enable a separation between the sensing layer (the one facing skin and containing the electrodes) and the connection layer (with the LIG/Ag composite tracks). Connection tracks are, thus, fully isolated with respect to the skin; also this strategy reduces the risk of contamination of the sensing electrodes during manufacturing. Such a design facilitates the stable connection to external acquisition devices, often problematic in thin/ultrathin wearable electrodes and epidermal electronics.^45,46^ The LIG VIAs had a resistance of R_VIAs_ = (7 ± 5) Ω and a diameter of ø_VIAs_ = (56 ± 11) *μ*m (see Fig. S6).

**FIG. 3.**
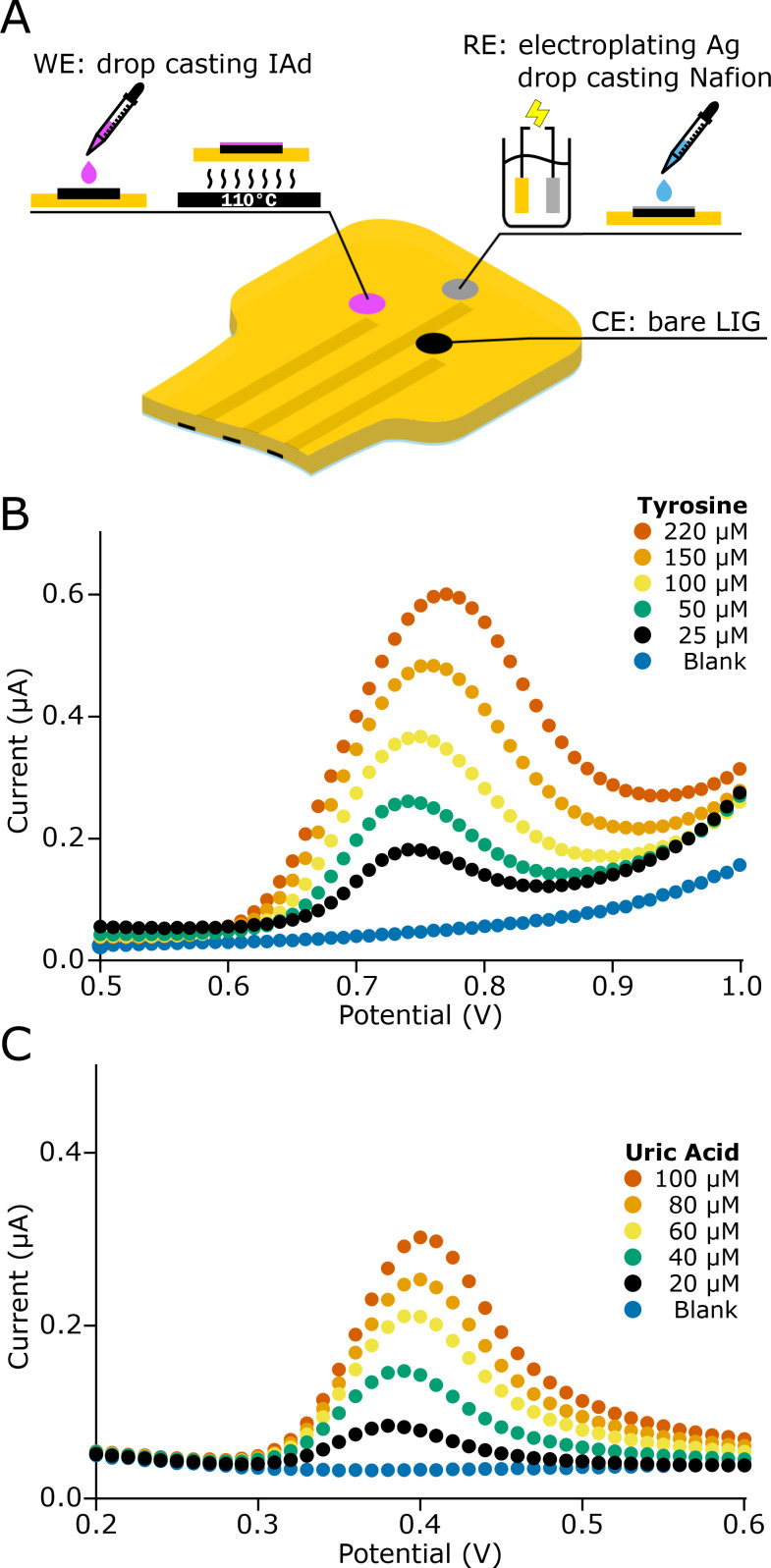
(a) Fabrication of the WE by functionalization of the LIG electrode with IAd and fabrication of the RE by electroplating with Ag and drop-casting of Nafion. (b) Square wave voltammetry (SWV) for the determination of tyrosine (25–220 *μ*M) and (c) SWV for the determination of uric acid (20–100 *μ*M).

When fabricating the sensor platform, one LIG electrode (WE) was functionalized with IAd, a second one (RE) was electroplated with Ag, treated with NaOCl and then coated with Nafion by drop-casting, whereas the third bare electrode was used as the counter electrode (CE) [Fig. 3(a)].

Figure S7 shows SEM images of the RE: there is a uniform distribution of Ag particles after the electroplating [Fig. S7(a)], whereas the treatment with an aqueous solution of NaOCl induces the formation of larger Ag particles and NaCl crystals [Fig. S7(b)]. The EDS analysis showed the formation of an outer AgCl layer enclosing the Ag particles [Figs. S7(c) and S7(d)].

The electrochemical platform was calibrated for each analyte at physiological concentration values in sweat. The tyrosine concentration resulted from the variation in the peak current at 0.75 V in the concentration range of 25–220 *μ*M [Fig. 3(b)]. Similarly, the uric acid concentration was determined in the range (20–100 *μ*M) from the peak current at 0.4 V [Fig. 3(c)].

As reported by deBethune *et al.*,^47^ the ionic conductivity in sweat can be related to the concentration of potassium, sodium, and chloride ions (the main ionic components), and thus, impedance was measured as a function of potassium and chloride ion concentration in the range 5–100 mM. The measurement of pH was determined as the shift in the peak position in the range of pH 4–10 related to the electrochemical reaction of the IAd (Fig. S8).

**FIG. 4.**
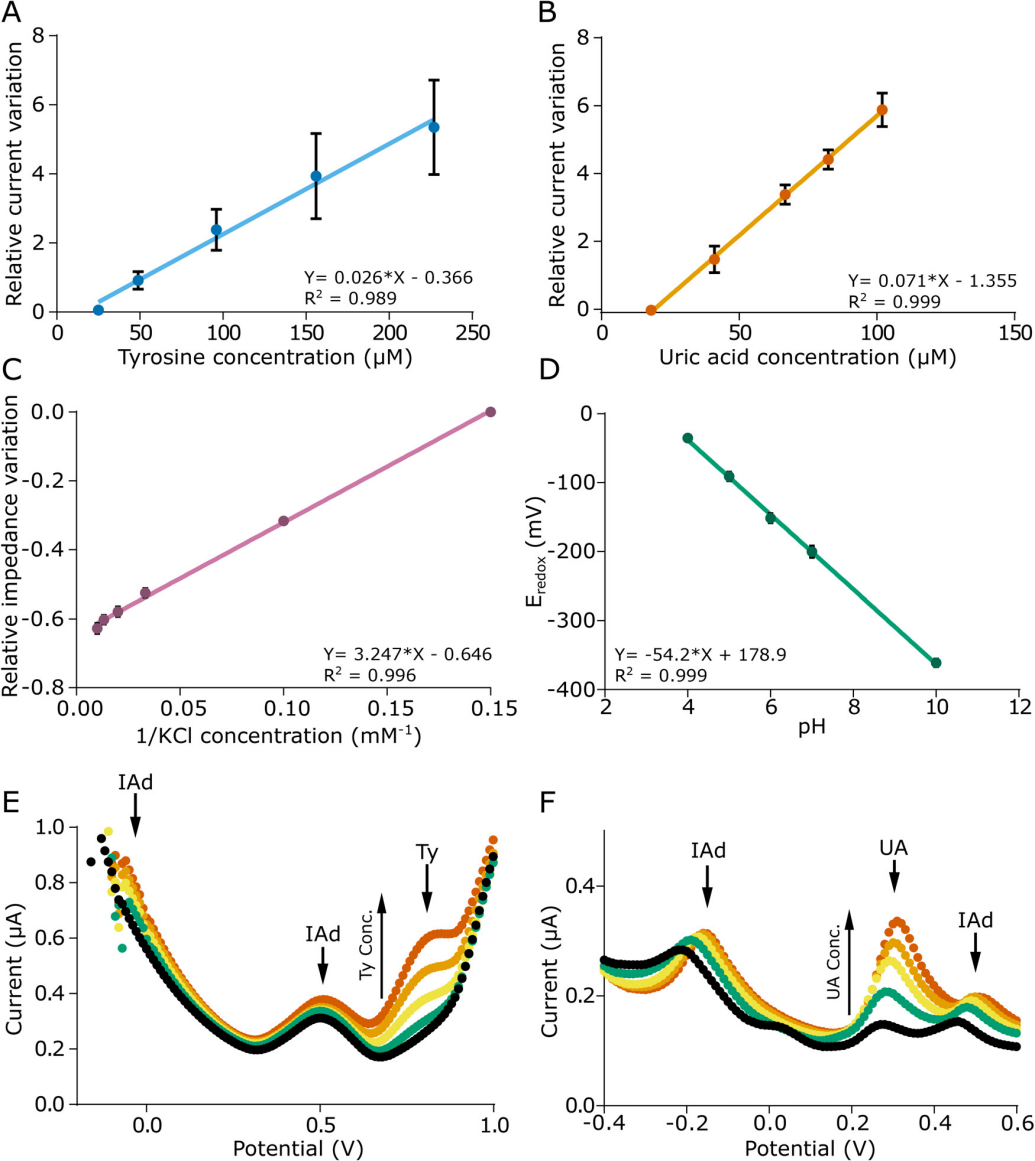
(a) Calibration curve for tyrosine. (b) Calibration curve for uric acid. (c) Calibration curve for different potassium and chloride ion concentrations (5–100) mM. (d) Calibration curve for pH sensing in the pH range of 4–10. (e) Effect of IAd on tyrosine response for different concentrations of tyrosine [concentrations as in (a)]. (f) Effect of IAd on uric acid response for different concentrations of uric acid [concentrations as in (b)]. Each point represents the average of at least three sensors measured in triplicate (n = 9).

The peak current at 0.75 V varied linearly with the tyrosine concentrations [Fig. 4(a)] in the whole range under investigation, with a sensitivity of 0.026 *μ*M^−1^ and 6% reproducibility defined as the relative standard deviation of the slope of the average calibration curve obtained with three sensors. It should be noted that the reported error is affected by the inter-sensor differences, which is higher at the potential of oxidation of tyrosine due to the beginning of the water evolution, whose intensity may change due to the different nature of each LIG electrode. This assumption is corroborated by the analysis of the calibration curve of a single sensor, shown in Fig. S9. Similar to tyrosine, a linear behavior was recorded for the uric acid calibration curve, with a sensitivity of 0.071 *μ*M^−1^ and 1% reproducibility [Fig. 4(b)].

Impedance was linearly correlated with the inverse of the ionic concentration [Fig. 4(c)]. Information on the LIG tracks can also be obtained from this curve. In fact, the real part of the impedance at 50 kHz is the sum of two different contributions as follows:

$$Z_{\mathrm{cell}}=Z_{\mathrm{ions}}+Z_{el},$$ (2)

where Z_ions_ is the impedance caused by the ions in solution, and Z_el_ is the impedance of the LIG tracks and electrodes. The increasing concentration of ions decreases the Z_ion_ contribution but has no effect on the Z_el_, whose value can be extrapolated from the intercept of the calibration curve.

A linear calibration curve was obtained for pH with an excellent sensitivity of −54.2 mV/pH [Fig. 4(d)], which is very similar to the Nernstian limit at 25 °C (−59 mV/pH).

Since the presence of IAd on the electrode surface may hinder or interfere with the electrochemical reactions of tyrosine and uric acid, voltammetric measurements at increasing concentrations [same levels of calibration, see Figs. 4(a) and 4(b)] of these analytes were performed with an excess of IAd [Figs. 4(e) and 4(f)]. In both cases, the peaks related to tyrosine and UA were visible and no significant effect on the calibration curve was recorded. IAd showed an additional peak (@ 0.5 V) to the one at −0.2 V, independently of the pH, which could be related to a secondary electron transfer of the compound.

A negligible effect of temperature T on the slope of the calibration curve was observed for each of the investigated analytes in the range of 4 < T < 40 °C (Fig. S10). Figure S11 reports the effect of high temperature on the height and positioning of the IAd peak. After the first thermal cycle (110 °C, 1 h), a shift in the potential was recorded, but the peak current did not change. This can be explained by a rearrangement of the RE during thermal curing, as the subsequent cycle showed no effect on the sensor response. We concluded that temperature may play a key role in RE stabilization. For this reason, all electrodes were cured at 110 °C upon fabrication.

### Biofluid sampling on skin model, analysis, and wearable application

**FIG. 5.**
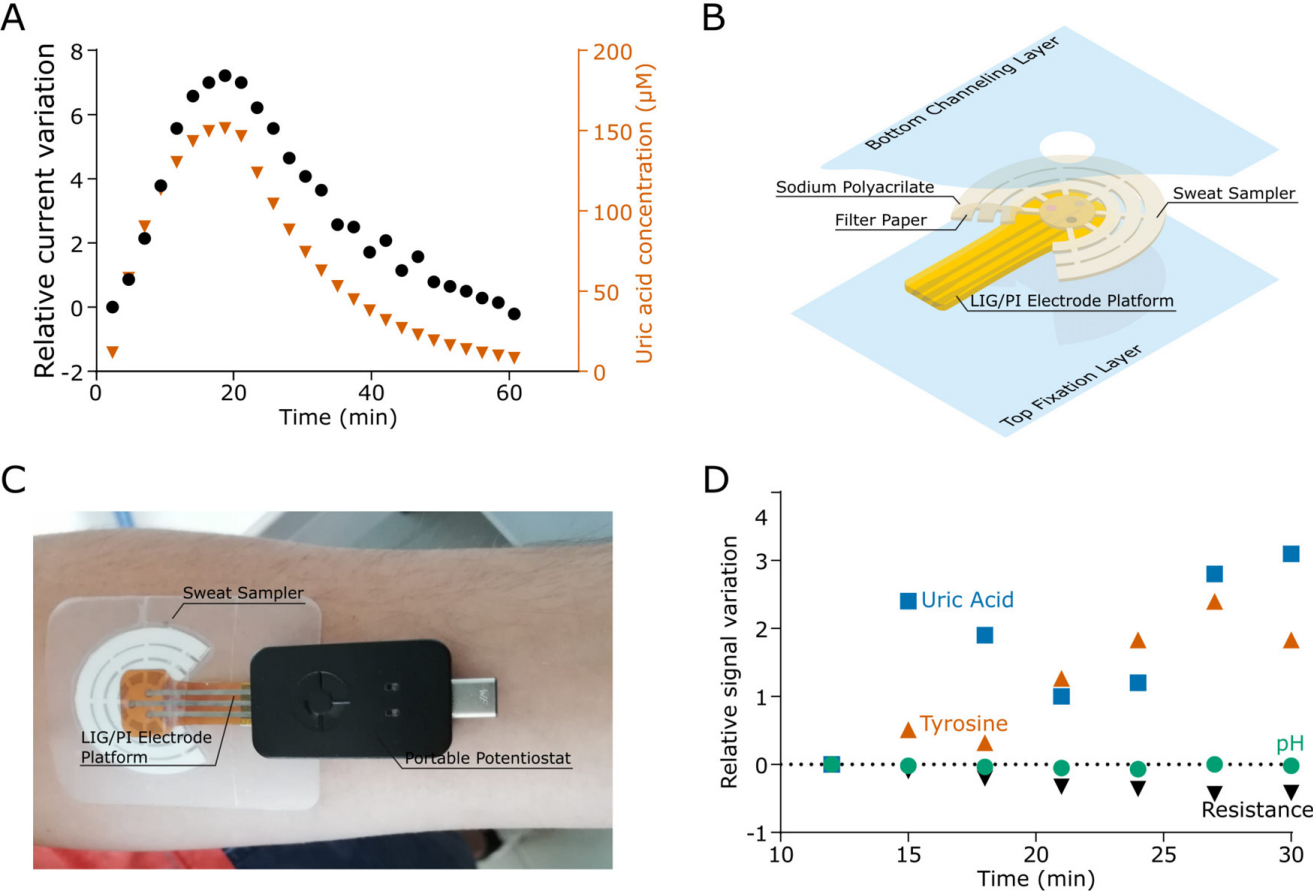
(a) Change in the sensing current for a time-dependent concentration of uric acid measured with the electrochemical platform. (b) Exploded schematics of the assembled wearable sweat sensing device. (c) Image of the device worn on skin and connected to a portable potentiostat. (d) Relative signal variation of uric acid, tyrosine, pH, and electrical impedance recorded by the device worn on a healthy subject, within 30 min of the sports activity.

In order to verify the behavior of the device under flow conditions, two peristaltic pumps were used to enable the sampler to collect a fluid with a time-dependent concentration of analytes. The response of the sensor, defined as the relative current variation of the peak at 0.4 V, was in good agreement with the calculated concentration [Fig. 5(a)], thus proving the ability of the device to work under variable concentrations of analytes in active flow conditions.

The uptake capacity of the paper sampler was evaluated by weighing the sampler before and after the addition of water, using a peristaltic pump with the flow rate equal to 5.8 *μ*l/min. The average capacity of the sampler was 57 ± 2 *μ*l (n = 9) for the bare paper design. The addition of the hygroscopic sodium polyacrylate powder increased the capacity to 470 ± 20 *μ*l (n = 6). The uptake speed was tested with a bare sampler at a flow rate of 1 *μ*l/min, which simulates the collection from an area of 78 mm^2^ in contact with skin sweating at a very high sweat rate (15 gm^−2^ min^−1^).^48^ The paper sampler took 1 h to fill at this flow rate, which confirmed its possible use for long-time measurements.

The assembled sensor prototype was attached onto a semi-solid agar plate (i.e., a phantom mimicking human skin and sweat) and EIS and SWV analyses were then performed. Within 10 s, a stable electrical contact was achieved through the LIG electrode/agar plate interface, confirming the successful sampling of liquids by the paper sampler underneath. Figure S12 shows the recorded SWV. The electrical resistance was stable throughout the measurement, thus proving the stability of the electrical contact. At the same time, the voltammetric signals of the other three analytes were visible and well resolute.

The device [exploded schematics Fig. 5(b)] was further tested in a real case scenario. Figure 5(c) shows the final device applied on the forearm of a test subject connected to the portable potentiostat. Electrochemical data were acquired every 3 min to prevent errors caused by the depletion of the analyte in the diffusive layer on the electrode surfaces. Figure 5(d) shows the relative change in signals recorded during 30 min of activity. During the first 10 min, the signal was unstable due to the low amount of sweat in the sampler. However, as soon as a stable sweat flow was reached, the electrochemical measurements showed a decrease in the sweat resistance, stating of an increase in the electrolyte concentration. Similarly, an increase in the tyrosine and uric acid levels led to an increasing current variation. The overall pH value did not change throughout the measured timescale.

### Cytotoxicity

**FIG. 6.**
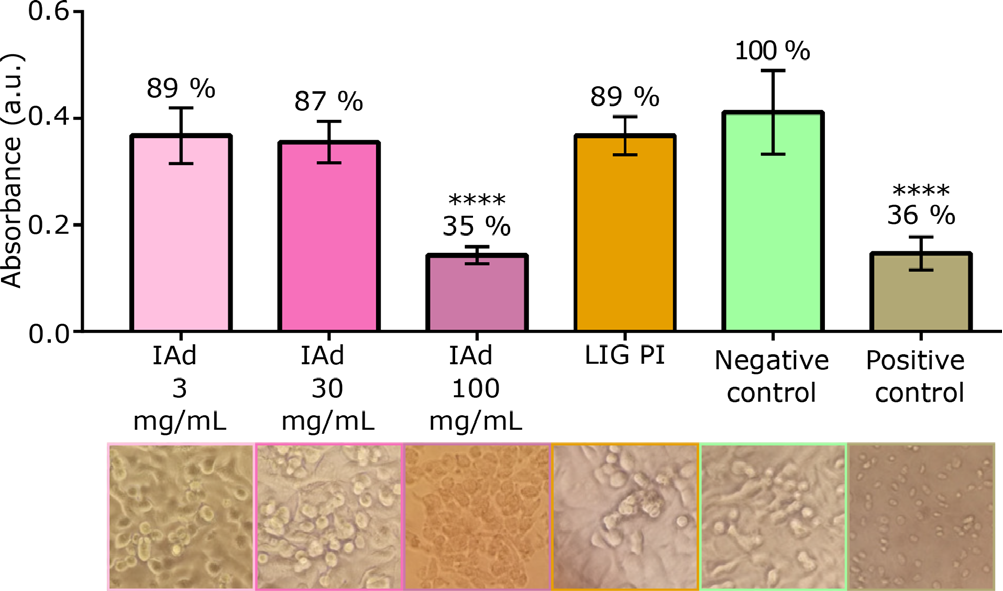
Absorbance values at 450 nm of formazan salt production as an approximation of the mitochondrial activity in A459 cells exposed to different concentrations of IAd ranging from 3 to 100 *μ*g/ml and LIG PI. The corresponding inverted microscope images (100×) are shown under the column bars. Culture media and 1% v/v Triton X-100 were included as negative and positive controls, respectively. The percentage of vitality shown for each condition (or treatment, material) was calculated considering the negative control as 100% viability. Data are expressed as means ± SD of three independent experiments with three replicates each ^****^P ≤ 0.0001.

The cytotoxicity of the IAd and PI/LIG used was investigated by XTT reduction assay of exposed cells. A549 cells were first exposed to different concentrations of IAd (3, 30, and 100 *μ*g/ml). Upon a 24 h incubation, a clear reduction in cell viability with increasing IAd concentrations was detected (Fig. 6). Particularly, at 100 *μ*g/ml IAd, a significant cell viability reduction was evident (p ≤ 0.0001) compared to the negative control, which instead showed similar absorbance values to those obtained for the positive control. On the other hand, exposure to 3 and 30 *μ*g/ml of IAd did not show any cytotoxic effect, as demonstrated by the percentage viability of 89% and 87%, respectively. Importantly, these values were not significantly different from the corresponding values of the negative control P = 0.06 and P = 0.19. The observation of the cellular morphology confirmed the cytotoxicity at elevated concentrations of IAd. In fact, cells exposed to 100 *μ*g/mL of IAd were found to lose the elongated shape characteristic of fibroblasts and instead presented a spherical shape (Fig. 6). Based on the ISO 10993-5:2009, 3 and 30 *μ*g/ml of IAd can be considered as non-cytotoxic. With 60 and 80 *μ*g/ml of IAd, cells showed a viability percentage equal to 77% and 58%, respectively (Fig. S13). Although both values were significantly different from the negative control, only 60 *μ*g/ml of IAd can be considered as non-cytotoxic according to the ISO 10993-5:2009 guidelines. The previous viability results were confirmed by the cellular shape. A549 cell exposure to LIG on PI demonstrated a non-cytotoxic effect, with a percentage of viability equal to 89%, which was not significantly different to the negative control (Fig. 6). Visualization under the microscope confirmed that cells kept the elongated shape. According to the ISO 10993–5:2009 guidelines, LIG PI is, thus, non-cytotoxic.

## CONCLUSIONS

This paper reports the development of a laser-scribed flexible and wearable device for sweat sampling and electrochemical analyses. The device consisted of two layers of adhesive PU embedding a paper sampler and LIG electrodes/tracks fabricated on PI. Two different 3D structures of LIG were investigated as possible electrodes for electrochemical measurements of sweat. The porous LIG structure proved to be the most suitable for the reported analyses. The device was calibrated for four different parameters, namely, pH, uric acid, and tyrosine concentrations and impedance as a function of ions (K^+^ and Cl^−^) in sweat. The multifunctional sensor was successfully tested on agar plates (skin phantom), and in a real case scenario, i.e., worn on skin during exercise. The cytotoxicity analysis showed negative results for each component of the device, proving its possible application without harm for the user. This work proved the possibility of fabricating a complete device capable of analyzing multiple analytes at the same time using a laser scribing fabrication approach, also highlighting several advantages compared to printing (wet) technologies.

The adoption of VIAs enables to separate the electrodes facing the skin from the connections to external devices, relieving drawbacks often encountered in other epidermal electronics. This permits to envision the development of multilayer devices, with separate stacked layers for sensing and communication including electronics, power sources, and antennas. We believe that the continuous progress of the scientific community in the development of LIG tracks on different substrates bodes well for the future development of the proposed device. LIG scribed on fully conformable and biodegradable substrates could increase both its wearability and environmental sustainability (use of renewable materials and disposal after end of use). Future studies will also focus on improving the LIG fabrication process in order to reduce the differences between the electroactive areas, and thus, the recorded current.

## METHODS

### Reagents and materials

Sodium hydroxide pellets (purity > 98%), monobasic potassium phosphate (purity > 99%), dibasic sodium phosphate dihydrate (purity > 99%), citric acid (purity > 99%), sodium tetraborate decahydrate (purity > 99%), sodium chloride (purity > 99%), potassium chloride (purity > 99%), hydroalcoholic solution of Nafion™ 5% w/w, l-tyrosine (purity > 99%), uric acid (purity > 99%), potassium hexacyanoferrate (purity > 99%), and sodium polyacrylate were purchased from Sigma Aldrich.

### LIG electrode fabrication

The LIG tracks and electrodes (∅ = 2 mm) were laser scribed onto polyimide (PI) sheets (Kapton HN, thickness = 50 *μ*m, supplied by RS Components Handelsgesellschaft m.b.H.) with a laser cutter/engraver (Universal Laser Systems VLS 2.30, Power 30 W). The system was operated with a CO_2_ laser source at 10.6 *μ*m wavelength and was equipped with an HPDFO (High Power Density Focusing Optics) beam collimator (nominal beam size in focus: 25.4 *μ*m). The scribing was performed in raster mode through the universal laser system interface and in an ambient air environment.

The settings for the produced LIG (porous LIG, LIG-P) were P = 10%, S = 10%, raster resolution of 500 PPI, image density (ID) of 5 (arbitrary scale, defining a spacing between consecutive rastered lines of ∼50 *μ*m), and a positive defocusing of Z = 0.7 mm.^29^

The settings for the preliminary investigated fibrous LIG (LIG-F) were P = 20%, S = 10%, raster resolution of 500 PPI, ID = 5, and Z = 0.7 mm.

The PI was cut to shape with the laser cutter. The carbonized cutting edges were cleaned by an ultrasonic bath (∼1 h) in a mixture of distilled water and isopropanol.

**FIG. 7.**
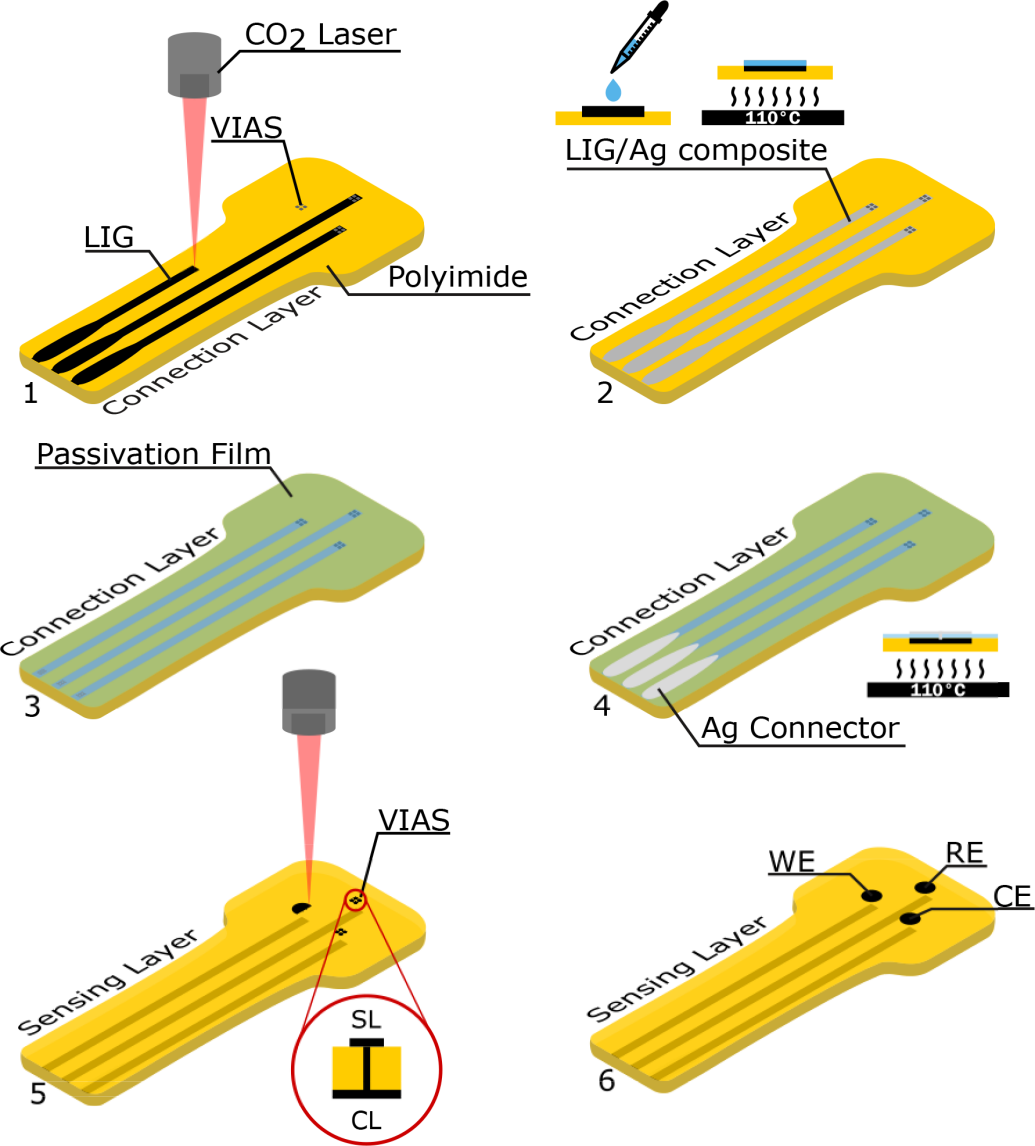
Manufacturing steps for LIG electrode platform: (1) scribing of the LIG tracks on one side of PI, laser cutting of holes for VIAs, (2) drop-casting reactive Ag ink and heating to create LIG/Ag composite on tracks, (3) and (4) assembling of passivation film (medical PU) with Ag connectors, and (5) final laser scribing of the LIG sensing electrodes on the other side of the PI. Inset shows the connection of the sensing layer (SL) with the connection layer (CL) with VIAS from LIG. (6) Finished LIG electrode platform showing the working (WE), reference (RE), and counter electrode (CE).

The cut out PI pieces were further processed by scribing the LIG tracks onto one side of the PI. The scribed tracks were modified with reactive silver ink by drop-casting and heated to 110 °C on a heating plate for 5 min to create a LIG/Ag composite with enhanced conductivity.^49,50^ By scribing and modifying the tracks before scribing the electrodes, it is important to ensure that the electrodes are not contaminated by (chemical) modifications of the tracks. After scribing the electrodes and LIG Vertical Interconnect Accesses (VIAs), which are used to connect the electrodes to the connection layer (side with LIG tracks), a sheet of adhesive medical polyurethane (PU) (Fixomull by BSN medical, properties listed in Table S1) with laser scribed perforations on the LIG tracks was placed on top of the connection layer. These perforations were coated with conductive silver ink (Engineered Conductive Materials, CI-1036) and dried on a heating plate at 100 °C for 5 min. The silver pads were used as connectors to the external potentiostat. Figure 7 shows the fabrication steps required. The dimension of the scribed LIG patterns can be found in Fig. S14.

### LIG characterization

The LIG morphology was investigated by a JEOL JSM-6490LV scanning electron microscope (SEM) operating at 5 kV acceleration voltage. LIG topography was investigated by atomic force microscopy (AFM) using a multimode 8 microscope (Bruker) operated in a peak-force tapping mode in air, equipped with silicon nitride cantilevers mounting single crystal silicon tips, with a nominal 8–12 nm radius, resonance frequency in the range 50–90 kHz, and force constant k = 0.4 N/m. Several 20 × 10 *μ*m^2^ AFM images were acquired with a scan rate of 1 Hz and sampling resolution of 1024 × 512 points. The images were flattened using line-by-line subtraction of first and second order polynomials. The Raman spectra were measured using a LabRam HR800 combined with an Olympus BX41 microscope with a laser at a wavelength of 352 nm (5 mW).

Profilometry measurements were performed with an AlphaStep D-500 profilometer from KLA-Tencor. The thickness of the pyrolyzed LIG was estimated by removing the LIG with adhesive tape and measuring the engraved step height on the precursor PI sheet between the lasered and non-lasered areas.

Contact angle measurements were conducted using a sessile drop technique, with a setup by KSV Instruments Ltd, using a CAM 200 optical contact angle meter. A drop of de-ionized water was placed on the surface of interest, and the contact angle was calculated with the KSV CAM2008 software. The drop volume was kept at around 10 *μ*l.

X-ray photoelectron spectroscopy (XPS) measurements were performed in an ultrahigh vacuum (UHV) chamber equipped with a dual anode x-ray source (Al/Mg) and a hemispherical electron energy analyzer (SPECS Phoibos 150). The measurements reported in this work were acquired with Al K alpha radiation (400 W). Both low resolution survey scans and high resolution detail scans of the regions of interest (C 1s and O 1s) were taken at normal emission. Quantification of the carbon and oxygen content was performed within a Prodigy software (SPECS).

Gas adsorption measurements were performed employing a Gemini surface area analyzer (Micromeritics, model 2365) on 0.5 cm large strips and several 1 cm long of LIG samples, following Borghi *et al.*^51^ Before each measurement, samples were degassed under a constant helium flux at 140 °C overnight using a dedicated unit to remove any possible contaminant absorbed by the pores of the material. During adsorption analysis, the relative pressure (P/P_0_) ranged from 0.05 to 0.99 for the acquisition of the isotherm plot. The specific surface area ( $A_{\mathrm{BET}}$, expressed in $m^{2}$/g) was calculated considering the relative pressure interval between 0.05 and 0.25. Pore size was estimated by the Barrett, Joyner, and Halenda (BJH) method^52^ on the adsorption isotherm.

The bending durability tests were executed with an electromechanical tensile/compression testing setup reported by Dallinger *et al.*^29^ Briefly, a computer controlled stepper motor induced the bending and a Keithley 2601B source meter sourcing 10 mA measured the change in resistance. The electrical resistance of LIG tracks scribed on PI [three samples, LIG dimensions: (40 × 1) mm] was recorded during 100 cycles of induced compression (30%) which resulted in a bending radius of 7 mm.

### LIG electrochemical platform

The electrodes were electrochemically activated by applying a voltage of −2 for 30 s vs a Pt rod in a 0.1 M KCl solution thanks to a DC power output (E3631A from Agilent). Different treatments, then, provided the working electrode (WE), reference electrode (RE), and counter electrode (CE) of the electrochemical platform.

The RE was prepared using the same setup by electroplating a silver layer on LIG with a voltage equal to −1.5 V for 30 s in a 0.1 M AgNO_3_ solution. The thin Ag layer was further treated with a 3% NaOCl aqueous solution for 1 h in order to form an AgCl layer. Finally, 3 *μ*l of Nafion 5% hydroalcoholic solution was deposited by drop-casting on the RE to prevent Ag oxidation at basic pH.

Sensitivity to pH of the WE was induced by drop-casting 1 *μ*l of a solution 3 *μ*g/ml of an indoaniline derivative (IAd)^53^ on its surface. Finally, the WE was cured at 110 °C for 1 h. No further modification was necessary for the measurement of sweat resistance, tyrosine, and uric acid concentrations. A bare LIG electrode was used as a CE.

### Sweat sampler fabrication

Filter paper (60 g/m^2^ from Filtros Anoia) was used to fabricate a sweat sampler in which five lines connected a central sampling zone (Ø = 10 mm) to reservoirs (Fig. S1). First, the sampler was laser cut with the scribing setup described previously (P = 23.5%, S = 100%, raster, 500 PPI, image density (ID) = 5, focused position). It was then integrated with the LIG platform into the final device using an adhesive medical polyurethane film (PU) that had also been laser cut into shape (P = 50%, S = 13%, raster, 600 PPI, image density (ID) = 5, positive defocus Z = 2.0 mm).

Sodium polyacrylate powder (100 mg) was embedded between the paper sampler and the adhesive PU layers to absorb sweat. The placement of the powder between the layers was chosen to avoid any direct contact with the skin [see Fig. 5(b)] and to only interact with the sweat after it has been analyzed by the electrochemical platform. The maximum capacity and the uptake speed of the sweat sampler were determined using a peristaltic pump P-1 (Pharmacia Fine Chemicals). For the capacity experiment, the pump flow was set to 2.6 ml/min, whereas a pump flow of 1.1 ml/min was used to determine the uptake speed.

### Validation of the electrochemical platform

Electrochemical measurements were performed by a Palmsens 4 potentiostat (Palmsens, The Netherlands) using PStrace 5.8 as a control software. Cyclic voltammetry in 0.1 M KCl solution on bare LIG electrodes was performed between −0.4 and −2.0 V with a scan rate of 0.1 V/s. Cyclic voltammetry between −0.2 and 0.8 V with a scan rate of 0.1 V/s was performed on bare and reduced LIG using 5 mM potassium hexacyanoferrate as a redox probe in 0.1 M phosphate buffer saline (PBS). The electroactive area of the LIG electrode was determined by performing cyclic voltammetry between −0.3 and 0.5 V with 5 mM potassium hexacyanoferrate at different scan rates (0.01, 0.03, 0.05, 0.1, 0.3, and 0.5 V/s). Tyrosine, uric acid, and pH were analyzed by means of square wave voltammetry (SWV) using the following method parameters: E_begin_ = −0.5 V, E_end_ = 1.0 V, Estep = 0.01 V, E_amplitude_ = 0.025 V, and f = 20 Hz. Resistance was measured using fixed frequency electrochemical impedance spectroscopy (EIS) using 0 V as DC potential, 25 mV as AC potential, 50 kHz as frequency sampling one point per second for a total of 10 s. Each measurement was performed in triplicate. All the reported potentials refer to the Ag/AgCl pseudo reference electrode fabricated on the LIG platform.

### Device performance

To verify the behavior of the device under flow condition, two peristaltic pumps were connected in series and used to add to the sampler a time-dependent concentration of uric acid following the scheme in Fig. S15. The amount of UA on the sampler was monitored with the LIG electrochemical platform by measuring relative current variation of the peak at 0.4 V obtained through SWV. Semisolid agar was used as a skin model^54,55^ to assess the performance of the sensing device. Plates containing 0.75% bacteriological agar (PanReac) were prepared in citrate buffer pH 6, adjusted with 20 mM NaCl to obtain an ionic strength value of 0.1 M. Tyrosine (50 *μ*M) and uric acid (50 *μ*M) were added. The agar solution was melted on a heating plate at 100 °C for 15 min, and then 7 ml were cast into Petri dishes (60 mm diameter) and stored at 4 °C until use. The device was additionally tested during a 30 min workout session. A Sensit Smart (Palmsens, The Netherlands) portable potentiostat was used for the electrochemical analyses.

### Cell cytotoxicity assessment

#### Cell culturing

Human lung carcinoma epithelial-like A549 cell line (ATCC-CCL 185) (LGC Standards, Sesto San Giovanni, Italy) was cultured in tissue culture flasks in Dulbecco's modified Eagle's medium (DMEM) (Euroclone) supplemented with 2 mM L-glutamine, 100 U/ml penicillin, 100 *μ*g/ml streptomycin, and 10% heat-inactivated Fetal Bovine Serum (Euroclone). Cell cultures were maintained at 37 °C in a 5% CO_2_ atmosphere. When cells grew to confluence, they were treated with 1× Trypsin-EDTA solution (Euroclone) and split in new flasks.

#### XTT reduction assay on the A549 cell line

In vitro cytotoxicity of IAd and LIG on PI were tested using the XTT reduction assay^56^ as recommended by ISO 10993–5:2009 for the biological evaluation of medical devices.^57^ This assay is based on the sodium 3′-[1-phenyl-aminocarbonyl)-3,4-tetrazolium]bis(4-methoxy-6-nitro)benzenesulphonic acid (XTT) with N-methylphenazonium methyl sulfate (PMS) as an electron coupling agent. XTT reduction into a formazan is measured spectrophotometrically, and the product color intensity can be correlated with the number of viable cells.

Prior to cytotoxicity determination, the IAd compound was prepared in DMEM and sterilized by filtration through a 0.22 *μ*m filter (Euroclone), while 12 mm^2^ of LIG PI samples were sterilized by an autoclave.

Cells cultured as previously described were resuspended in complete DMEM, then 100 *μ*l of the cell suspension were seeded to a concentration of 10^4^ cells/well in flat-bottom 96-well microtiter plates and incubated for 24 h at 37 °C in a 5% CO_2_ atmosphere. The following day, the culture media was removed and replaced with 100 *μ*l of fresh complete DMEM containing the appropriate concentrations of IAd to be tested (3, 30, 60, 80, and 100 *μ*g/ml) or complete DMEM alone for solid materials (LIG PI), which were then gently placed on the cell layer. Culture media alone and 1% (v/v) Triton X-100 (Sigma) were also included as negative and positive controls, respectively. After a 24 h incubation at 37 °C in a 5% CO_2_ atmosphere, the culture media was removed and 100 *μ*l of phosphate buffered saline (PBS) 1× was added. A total of 50 *μ*l of freshly prepared XTT/PMS solution was then added and the plate was incubated for 1 h at 37 °C in a 5% CO_2_ atmosphere protected from the light. Finally, 100 *μ*l of the solution was transferred into a new plate and absorbance at 450 nm was read using a microplate reader (Multiscan GO, Thermo Fisher Scientific). Three independent experiments with four replicates each were performed.

Cytotoxicity was presented as the percentage of cell viability with respect to the negative control, and calculated as follows:

$$\%\mathrm{Cell}\;\mathrm{viability}=\frac{\mathrm{OD}\;450s}{\mathrm{OD}\;450n}\times 100,$$

where

OD 450s is the absorbance at 450 nm of formazan salt production by A549 cells upon exposure to the test sample.

OD 450n is the absorbance at 450 nm of formazan salt production by A549 cells in the negative control.

The lower the viability value the higher the cytotoxic potential of the compound or material.

If viability is reduced to < 70% with respect to the blank the test sample is considered as having a cytotoxic potential.

#### Statistical analysis

Data were expressed as mean ± SD. Statistical analysis was performed using ANOVA followed by Dunnett's multiple comparison test, with a significance level of P ≤ 0.05 using the GraphPad Instat software (v. 6.05 for Windows, La Jolla, CA, United States).

## SUPPLEMENTARY MATERIAL

See the supplementary material for details the additional information including electrochemical measurements, SEM images, electromechanical measurements, EDX analysis, and cytotoxicity investigations.

## Data Availability

The data that support the findings of this study are available from the corresponding authors upon reasonable request.
